# Why Does Therapy Work? An Idiographic Approach to Explore Mechanisms of Change Over the Course of Psychotherapy Using Digital Assessments

**DOI:** 10.3389/fpsyg.2020.00782

**Published:** 2020-04-24

**Authors:** Allison Diamond Altman, Lauren A. Shapiro, Aaron J. Fisher

**Affiliations:** ^1^Idiographic Dynamic Lab, Psychology Department, University of California, Berkeley, Berkeley, CA, United States; ^2^Psychology Department, The Wright Institute, Berkeley, CA, United States

**Keywords:** case study, digital assessments, mechanisms, cognitive behavioral therapy, ambulatory assessment

## Abstract

**Background and Objective(s):**

While psychotherapy treatments are largely effective, the processes and mechanisms underlying such positive changes remain somewhat unknown. Focusing on a single participant from a treatment outcome study that used a modular-based cognitive behavior therapy protocol, this article aims to answer this question by identifying changes in specific symptomatology over the course of the treatment. Using quantitative data derived from digital health methodology, we analyzed whether a given therapeutic intervention was related to downstream effects in predicted symptom domains, to assess the accuracy of our interventions.

**Methods:**

This case study employed an observational N-of-1 study design. The participant (*n* = 1) was a female in the age range of 25–35 years. Using digital health data from ambulatory assessment surveys completed prior to and during therapy, separate linear regression analyses were conducted to assess if hypothesized treatment targets reduced after a given module, or intervention.

**Results:**

Support was found for some of the hypothesized quantitative changes (e.g., decreases in avoidance after exposures module), yet not for others (e.g., decreases in rumination following the mindfulness module).

**Conclusion:**

We present data and results from our analyses to offer an example of a novel design that may allow for a greater understanding of the nature of symptom changes with increased granularity throughout the course of a psychological treatment from the use of digital health tools.

## Introduction

The field of clinical psychology has undergone many changes in the past decade. After the introduction of the Research Domain Criteria (RDoC; Insel et al., 2010) in 2010, researchers seeking external funding have been incentivized to move away from investigating psychopathological constructs at a disorder-level, in order to explore a dimensional system that encompasses multiple levels units and of analysis, from genes at the most basic, granular level to behavior at the most macroscopic. While the development of the RDoC was not intended to replace the Diagnostic and Statistical Manual of Mental Disorders (DSM-V) and serve as a diagnostic guide, its introduction nonetheless serves as a reminder that our current diagnostic system, and treatment development efforts, may be structurally flawed by the sheer heterogeneity underlying diagnostic labels as they currently stand. Taking the diagnosis of Posttraumatic Stress Disorder (PTSD) as an example, current DSM-5 criteria allow 636,120 combinations of presenting symptoms to exist in order to meet criteria for the diagnosis, meaning that it is possible for 636,120 individuals to meet criteria for PTSD, with no repeats in the exact constellation of symptoms from person to person (Galatzer-Levy and Bryant, 2013). Treatments for such conditions have been historically developed by researchers based on diagnostic categories and group-level (i.e., nomothetic) information. These often fail to produce significant change in a large subset of individuals, and outcomes for the treatment of many mental health conditions are lower than desired.

Over the past 30 years there has been a long history of researchers seeking to understand underlying mechanisms of psychotherapy success, and over time numerous models of change have emerged, including but not limited to psychotherapy integration (Stricker and Gold, 1996), the common factors approach (CF; Frank and Frank, 1991; Wampold, 2007), theoretical integration (Stricker and Gold, 1996), phase models (Howard et al., 1993), and the transtheoretical states of change model (Prochaska and DiClemente, 1983). From the psychotherapy integration approach, which aims to look beyond single approaches and instead hopes to integrate multiple perspectives, to the common factors approach, which proposes that different approaches in psychotherapy share common factors that account for the majority of the effectiveness of a psychological treatment, each model has developed their own ways of assessing and understanding change in psychotherapy. While these models indicate the efforts of researchers to understand why treatments may work, the majority of work in treatment development still focuses on groups, rather than individuals.

Conversely, clinicians typically focus on single patients, often using case conceptualization methods such as the case formulation approach to cognitive-behavior therapy (Persons, 2012). These often involve series of linked interventions typically following comprehensive assessments of the patient, and allow clinicians to choose techniques based on the best match to the presenting needs and problems of the patient.

To bridge the gap between research and practice, it is imperative for researchers to gather information that will be immediately useful to clinicians. Such information may come from idiographic treatment models, wherein researchers investigate change in individual patients, rather than diagnostic groups, to explore mechanisms of change over the course of a given treatment. Recently, and following along the footsteps of other medical domains such as oncology, there has been a push toward an idiographic, personalized approach to psychotherapy research, focusing on the precise symptomatology of an individual patient instead of broad diagnostic categories for generating treatment decisions. One such approach, outlined by Fisher (2015), calls for an idiographic, dynamic methodology whereby clinicians conduct person-specific dynamic assessments that yield information about syndrome structures and states to provide actionable information for personalized interventions. This work requires intensive, repeated digital assessment measures for individual patients, with the hope that this intensive measurement will yield prescriptive information for improved results. In a recent open trial of personalized modular CBT using the Unified Protocol (UP; Barlow et al., 2011), Fisher et al. (2019) demonstrated a Hedge’s *g* of 2.33 over an average of 10 sessions, outperforming an historical average effect size for from a recent-meta-analysis (Johnsen and Friborg, 2015). The UP is typically delivered over 16 sessions, and has not been shown to demonstrate equivalent effects in trials to date (c.f. Farchione et al., 2012). While additional work is required to substantiate such an intensive approach to personalization, this trial illustrates that treatment based off of idiosyncratic structures of psychopathology may be an important part of improving overall treatment efficacy in the mental health domain. This information then can be immediately useful to practicing clinicians hoping to understand how best to approach singular cases.

The following case study aims to use the same person-specific ambulatory assessment data to investigate changes occurring in an individual over the course of a treatment, focusing on a single participant from the Fisher et al.’s (2019) open trial—participant 007 (P007). The idiographic approach outlined by Fisher et al. (2019) involves intensive, repeated digital assessment measures, captured four-times-daily for approximately 30 days. This provides sufficient data to facilitate person-specific factor analyses and dynamic factor modeling. In the open trial, pre-therapy analyses were used to determine predominance among latent symptom clusters in order to generate targeted therapies (using existing modules of the UP) person by person. Participants were also given the chance to extend these surveys and continue to complete them throughout the course of therapy, as did P007. The current article aims to use P007’s data to identify changes in specific symptomatology over the course of treatment in order to identify if a given therapeutic intervention, or module (e.g., mindfulness) was related to downstream effects in predicted symptom domains (e.g., reduced restlessness). It should be acknowledged that some researchers have proposed that efficacy of psychotherapy is not due to specific interventions or techniques, but rather from factors of psychotherapy common to all treatments, referred to as common factors (Luborsky et al., 1975; Wampold, 2001, 2007). Yet, the current research aims to define specific effects that can be attributed to certain interventions, rather than common factors. This novel design may allow for a greater understanding of the nature of symptom changes with increased granularity throughout the course of treatment, and may serve as a model for clinicians and researchers to incorporate such work in their own research and practice.

As noted above, in the treatment trial from which P007’s data was draw, delivery of the UP was personalized based on data gathered prior to therapy, which was then subjected to an analysis for the identification of latent symptom dimensions and dynamic factor modeling to determine the dynamics and module delivery order (see Fernandez et al., 2017; Fisher et al., 2019). Each individual patient received a specific delivery order of the modules based on their presenting symptoms and relationships among symptom dimensions. The module order for P007 is outlined in Table 1. For the present study, hypotheses were developed based on specific modules in order to investigate symptom changes throughout therapy, with expected changes to appear after a given module was delivered. Each hypothesis is outlined in Table 2 and briefly reviewed below.

**TABLE 1:** Sessions and module orders for P007.

Session(s)	Therapy module
1	Motivation and enhancement
2	Emotional awareness and tracking
3	Emotional awareness and tracking
4	Mindfulness
5	Mindfulness and non-judgmental awareness
6	Mindfulness and non-judgmental awareness
7	Exposures (imaginal and *in vivo*)
8	Exposures (imaginal and *in vivo*)
9	Cognitive appraisals and reappraisals
10	Cognitive appraisals and reappraisals
11	Emotion-driven behaviors and emotional avoidance
12	Emotion-driven behaviors and emotional avoidance
13	Relapse prevention
14	Relapse prevention

**TABLE 2:** Module-specific hypothesized relationships in digital assessment survey data over the course of therapy.

Therapy module	Quantitative survey hypothesis
Pre-therapy period	N/A
Motivation enhancement	N/A
Emotional awareness and tracking	N/A
Mindfulness and non-judgmental awareness	Reduced restlessness, dwelling on the past, worry; reduced worthlessness and guilt
Exposures (imaginal and *in vivo*)	Reduced difficulty concentrating, avoiding activities, avoiding people, procrastination, reassurance seeking
Cognitive appraisals and reappraisals	Reduced worry, depression; increased positivity, contentedness
Emotion driven behaviors and emotional avoidance	Reduced worry, depression
Relapse prevention	N/A

Prior to delivery of the first module, the participant underwent a 30-day pre-therapy assessment. Although this data collection was intended to reflect stationary processes, engagement with treatment providers can have distress-reducing effects for dysphoric individuals. Therefore, stationarity, the assumption that the mean, variance, and auto-correlation remain relatively stable over time, may be violated because of a process known as remoralization (Howard et al., 1993). Howard’s remoralization theory (1993), proposes that psychotherapy entails sequential changes and the first change is an enhancement in the patient’s sense of subjective well-being, which typically occurs before the process of formal psychotherapy beings (Howard et al., 1993). In this study, remoralization may have occurred during engagement with the phone surveys prior to the start of treatment delivery. While no formal hypotheses were made during this initial pre-therapy period, data from it is included in this study.

Therapy began with an emotional awareness and tracking module, and no significant changes were expected after this module, as the intervention was primarily targeting overall emotional awareness across both positive and negative affect domains. Because this relates to processes already in place from the pre-therapy assessment, we believed that – to the degree that self-monitoring may elicit symptomatic change – these changes would have already occurred.

Hypothesis 1: The second module delivered was a mindfulness module, and it was hypothesized that after the delivery of this module the participant would report reductions in feelings of restlessness and dwelling on the past on their survey responses. Extant work across a variety of domains has illustrated the success of a mindfulness-based approach in reducing physiological restlessness, including using a mindfulness-based stress reduction (MBSR) paradigm to reduce symptoms of restless leg syndrome (Bablas et al., 2016) and using a MBSR approach to reduce levels of pre-sleep arousal (Cincotta et al., 2010). Extant work in the literature has similarly demonstrated a negative correlation between mindfulness and rumination (Jain et al., 2007; Svendsen et al., 2017), hence we expected reductions in the survey item “dwelled on the past” following delivery of this module.
Hypothesis 2: The third module was an *in vivo* exposure intervention, aimed at facilitating habituation and inhibition of fear-conditioning. We hypothesized that the participant would report reductions in avoidance-related items (avoiding people, avoiding activities, procrastinating, and seeking reassurance) after delivery of this module, based on an abundance of previous work illustrating the effect of exposures on reducing avoidance (e.g., Foa and Kozak, 1986).
Hypothesis 3: The fourth module was a cognitive appraisal and reappraisals module, and it was hypothesized that after delivery the participant would report reductions in worry, and depression, and increases in positivity and contentedness, as reappraisals have previously been shown to reduced symptoms of stress and stress-related symptoms (Moore et al., 2008).
Hypothesis 4: Lastly, the fifth and final module was an emotion driven behaviors and emotional avoidance module. It was hypothesized that after this module, the participant would report further reductions in feelings of worry and depression, as a large body of work has indicated reductions in anxiety and depressive symptoms following emotional exposures (Foa and Kozak, 1986; Hayes et al., 2005).

This quantitative, survey-based approach allows for an in-depth investigation and quantification of therapeutic changes across the course of a modularized individualized therapy in a single participant. We propose that this novel design will lend greater insight into individual symptom perturbations throughout the course of therapy.

## Materials and Methods

### Setting and Participant

Data was obtained as part of an ongoing research study at the University of California, Berkeley. The participant, a female in the age range of 25–35 years old, was initially recruited to participate in a multi-phase personalized treatment study, for which she completed an initial diagnostic assessment, 14 weeks of individualized therapy treatment, and phone surveys from after the diagnostic assessment through the conclusion of therapy. Inclusion criteria included the following: principal diagnosis of either GAD or MDD; no concurrent psychosocial treatment; the participant had not previously received CBT; no medical conditions were identified as contributors to anxiety problems (e.g., hypoglycemia, thyroid problems); and mania and/or psychosis were absent. All procedures of the study were conducted under the approval of the University of California, Berkeley Institutional Review Board. Additional demographic information can be found in Table 3.

**TABLE 3:** Demographic information for participant 007.

Age range	25–35
Gender	Female
Marital status	Single
Race/ethnicity	African American
Education level	Some college

### Procedure

The participant was initially recruited via paper and electronic advertisements placed in the community. After obtaining verbal consent, she completed a brief telephone screening, and based on this preliminary information, the participant was invited for an in-person structured clinical interview. They presented to the University of California, Berkeley’s Department of Psychology building for clinical assessment, during which the anxiety and related disorders interview schedule for DSM-5 (Brown and Barlow, 2014), Hamilton Anxiety Rating Scale (Hamilton, 1959), and Hamilton Rating Scale for Depression (Hamilton, 1960) were administered by an advanced graduate student in clinical psychology. Results were reviewed with a supervising psychologist before the participant was invited to enroll in the study. After consent paperwork was reviewed, the participant took part in a two-phase study: Phase 1 required the completion of daily surveys to assess mood and anxiety disorder symptoms, and Phase 2 involved a 14-week cognitive-behavioral therapy treatment based on The Unified Protocol for Transdiagnostic Treatment of Emotional Disorders (Barlow et al., 2011; details described in Fisher, 2015). During Phase 2, the participant was instructed to continue the daily surveys in order to track progress in treatment. In both phases of the study, the individual received four text messages per day, each one containing a hyperlink to a web-based survey, resulting in four surveys per day. P007 completed surveys for a total of 42 days during Phase 1 (pre-therapy) and 140 days during Phase 2 (during therapy), with 158 and 437 total viable, non-missing observations for Phase 1 and Phase 2, respectively. The participant completed 96% of their surveys throughout Phase 1, and 78% throughout Phase 2, with an overall compliance rate of 82%.

### Measures

• *Anxiety and Related Disorders Interview Schedule for DSM-5 (ADIS-5; Brown and Barlow, 2014)*. The ADIS-5 is a semi-structured clinical interview designed to diagnose current anxiety, mood, and related disorders according to new DSM-5 criteria. This updated version of the ADIS-5 builds upon previous versions, which all exhibited well-established reliability. The ADIS-5 demonstrates good-to-excellent interrater reliability for DSM-5 disorders (kappa ranging from 0.67 to 0.86, with the exception of dysthymia, kappa = 0.31) (DiNardo et al., 1994).
• *Hamilton Anxiety Rating Scale (HAM-A; Hamilton, 1959).* The HAM-A is a 14-item clinician administered scale that assesses severity of anxious symptoms. This scale provides a severity rating of each overarching symptom cluster on a scale from 0 (not present) to 4 (very severe). Research has shown that retest reliability for the HAM-A was good (intraclass correlation coefficient 0.86) and interrater reliability ranged from an intraclass correlation coefficient of 0.74–0.96 (Bruss et al., 1994). Construct validity has also been demonstrated in clinical samples (Beck and Steer, 1991).
• *Hamilton Depression Rating Scale (HAM-D; Hamilton, 1960).* The HAM-D is a 13-item clinician administered scale developed to assess the severity of depressive symptoms. This scale provides severity ratings of each overarching symptom cluster on a scale from 0 (not present) to 4 (very severe/incapacitating). Internal consistency of the HAM-D ranges from adequate to good (0.73–0.81; Steer et al., 1987; Moras et al., 1992). HAM-D have also been shown to correlate significantly with self-report measures of depression in clinical samples (Steer et al., 1983).
• *Digital Assessment Daily Survey Items.* In addition to the extant DSM-5 GAD and MDD symptom criterion, daily surveys included four behavioral symptoms: (a) avoiding activities with possible negative outcomes, (b) preparing for possible negative outcomes, (c) procrastinating about taking action or decision making, and (d) seeking reassurance, as recent data have illustrated these behavioral symptoms to be significant features of GAD and MDD phenomenology (Beesdo-Baum and Knappe, 2012). The participant rated their experience of each symptom domain over the preceding 4 h (the surveys were randomized to roughly a 4-h interval schedule) on a 0–100 visual analog slider, with anchors of *not at all* and *as much as possible* anchored at the 0 and 100 positions, respectively. Survey items are presented in Table 4.

**TABLE 4:** Daily digital assessment survey items.

**item**
1. To what degree have you felt energetic
2. To what degree have you felt enthusiastic
3. To what degree have you felt content
4. To what degree have you felt irritable
5. To what degree have you felt restless
6. To what degree have you felt worried
7. To what degree have you felt worthless or guilty
8. To what degree have you felt frightened or afraid
9. To what degree have you experienced loss of interest or pleasure
10. To what degree have you felt angry
11. To what degree have you felt hopeless
12. To what degree have you felt down or depressed
13. To what degree have you felt positive
14. To what degree have you felt fatigued
15. To what degree have you experienced muscle tension
16. To what degree have you had difficulty concentrating
17. To what degree have you felt accepted or supported
18. To what degree have you felt threatened; judged; or intimidated
19. To what degree have you dwelled on the past
20. To what degree have you avoided activities
21. To what degree have you sought reassurance
22. To what degree have you procrastinated
23. To what degree have you avoided people

### Statistical Approach to Quantitative Survey Items

Data for each specific module-based hypothesis was subset for individual hypotheses. For analyses done on each specified module, data from that module and throughout the rest of therapy was used in order to assess the degree to which each *specific* module, or intervention, was associated with changes in the specific hypothesized downstream variables. For example, the Exposures module was the 4th module for this participant, so data was subset from the date that module started through the last day of therapy for the exposure module-based hypotheses, and data prior to delivery of that module was excluded. To then assess changes in the participant’s survey responses over the course of therapy, ordinary least squares (OLS) linear regression was employed to test response trajectories of each item. That is, separate linear regressions were applied to test the relationship between Time (coded in days) and changes in the dependent variable in question (e.g., worry, rumination, procrastination). The decision to use OLS regressions for trends over time instead of a multilevel approach was chosen due to much prior work in our lab indicating that one can handle intraindividual temporal dependence equally well with trend or AR components.

## Results

For OLS regression analyses, rows of data with missing surveys were excluded as a function of listwise deletion. In order to explore the presence of remoralization as predicted by Howard’s theory, we tested the degree to which the client experienced reductions in negative-affect items and increases in positive-affect items. Thus, the data was subset into the portion of time prior to the start of therapy, and then separate linear regression analyses were employed to predict specific negative affect items as a function of time. Results are presented in Table 5. Models for negative affect items of “dwelled on the past,” “felt worthless or guilty,” “felt worried,” “felt irritable,” “experienced a loss of interest or pleasure,” “felt threatened, judged, or intimidated,” “felt down or depressed,” and “felt angry” were all significant at the *p* < 0.05 level, indicating significant decreases in these negative affect items during the pre-therapy period.

**TABLE 5:** Separate linear regression models for the trajectories of negative affect items as a function of time during the pre-therapy period.

	Negative Affect items over Time			
	*p*	SE	*t*	*p*
Dwelling on the past	–0.304	0.0275	–11.03	< 0.00*
Worthless or guilty	–0.10	0.04	–2.72	0.01*
Hopelessness	–0.07	0.04	–1.083	0.07
Worry	–0.21	0.03	–6.69	< 0.00*
Irritability	–0.23	0.03	–7.93	< 0.00*
Loss of interest or pleasure	–0.19	0.04	–5.18	< 0.00*
Threatened, judged, intimidated	–0.19	0.03	–6.18	< 0.00*
Down or depressed	–0.18	0.03	–6.66	< 0.00*
Anger	–0.10	0.04	2.79	0.01*

* indicates significance at *p* < 0.05.

Positive affect items were tested in the same manner, with separate simple linear regressions to test trajectories over time during the pre-therapy period. All positive affect items emerged as significant; during the pre-therapy period, feeling positive significantly increased over time [β = 0.30, F(1,156) = 176.4, *p* < 0.01, *R*^2^ = 0.53], feeling energetic significantly increased over time [β = 0.30, F(1,156) = 184, *p* < 0.01, *R*^2^ = 0.54], feeling enthusiastic significantly increased over time [β = 0.32, F(1,156) = 173.9, *p* < 0.01, *R*^2^ = 0.52], feeling content significantly increased over time [β = 0.33, F(1,156) = 154.6, *p* < 0.01, *R*^2^ = 0.49], and feeling accepted or supported significantly increased over time [β = 0.17, F(1,156) = 29.69, *p* < 0.01, *R*^2^ = 0.15].

In order to test our first hypothesis, that participant would report reductions in restlessness and rumination following the mindfulness interventions, the data was subset to the time period after the module was delivered, and separate linear regression analyses were employed to test the trajectory of feelings of restlessness and dwelling on the past over time. Results were significant for restlessness, yet in the opposite direction then hypothesized; after delivery of the module, the participant reported significantly increased feelings of restlessness [β = −0.08, F(1,167) = 8.43, *p* < 0.01, *R*^2^ = 0.04].

In order to test the hypothesis that P007 would report reductions in avoidance-related items (difficulty concentrating, avoiding people, avoiding activities, procrastinating, and seeking reassurance) following the exposure module, data was again subset for after the module was delivered, and separate linear regression analyses were employed to test the trajectory of each avoidance-related item over time. Significant reductions were observed for difficulty concentrating [β = −0.16, F(1,122) = 7.82, *p* = 0.01, *R*^2^ = 0.05], avoiding people [β = −0.39, F(1,122) = 34.93, *p* ≤ 0.01, *R*^2^ = 0.22], and procrastinating [β = −0.25, F(1,122) = 19.18, *p* ≤ 0.01, *R*^2^ = 0.13]. Seeking reassurance significantly increased over time following the exposures module [β = 0.18, F(1,122) = 7.88, *p* = 0.01, *R*^2^ = 0.05], which we hypothesize may be due to the participant conceptualizing reassurance seeking as a pro-social quality rather than a safety behavior (more in discussion).

In order to test the hypothesis that the participant would report reductions in worry and depression and increases in positivity and contentedness following the reappraisal module, the data was again subset for after the module was delivered, and separate linear regression analyses were employed to test the trajectory of worry, depression, positivity, and contentedness over time. No significant findings emerged from these models. However, it should be emphasized that this may be due to the embedded nature of these constructs in all modules and symptomatic experiences. Thus, as a secondary analysis, we examined the entire therapy section of the time series to assess the degree of change in depression, worry, positivity, and contentedness across the complete treatment period. Results indicate significant reductions in depression [β = −0.04, F(1,434) = 28.88, *p* ≤ 0.00, *R*^2^ = 0.06] and worry [β = −0.07, F(1,434) = 95.23, *p* ≤ 0.00, *R*^2^ = 0.18], and a significant increase in contentedness [β = 0.02, F(1,434) = 4.69, *p* = 0.03, *R*^2^ = 0.01] over the complete treatment period.

In order to test the final hypothesis, that the participant would report further reductions in feelings of worry and depression, the data was again subset for after the final module was delivered, and separate linear regression analyses were employed to test the trajectory of worry and depression over time. Feelings of worry significantly decreased over time following delivery of this module [β = −2.05, F(1,17) = 5.71, *p* = 0.03, *R*^2^ = 0.21]. No significant findings emerged for feelings of depression.

In addition to the module-specific hypotheses, we also employed separate linear regressions for each survey item as a function of time over the entire therapy period to the data. Results are depicted in Table 6. Significant reductions were observed for the following items: dwelling on the past, loss of interest or pleasure, procrastinated, and feeling worthless or guilty, hopeless, worried, irritable, threatened or judged, down or depressed, restless, fatigued, and energetic. Significant increases were observed for the following items: sought reassurance, feeling content, and feeling accepted or supported.

**TABLE 6:** Separate linear regression models for the trajectories of all survey items as a function of time during the entire therapy period.

	Change in all survey items over entire therapy *y* Period			
	*p*	SE	*t*	*p*
Dwelling on the past	–0.20	0.01	–4.22	< 0.00*
Worthless or guilty	–0.20	0.01	–4.22	< 0.00*
Hopelessness	–0.40	0.01	–8.98	< 0.00*
Worry	–0.42	0.01	–9.76	< 0.00*
Irritability	–0.28	0.01	–6.04	< 0.00*
Loss of interest or pleasure	0.06	0.01	1.27	0.21
Threatened or judged	–0.12	0.01	–2.51	0.01*
Down or depressed	–0.25	0.01	–5.37	< 0.00*
Anger	–0.02	0.01	–0.32	0.75
Frightened or afraid	–0.01	0.02	–0.15	0.88
Restless	–0.16	0.01	–3.44	0.00*
Fatigued	–0.18	0.01	–3.91	0.00*
Muscle tension	–0.08	0.01	–1.69	0.09
Difficulty concentrating	–0.41	0.01	–9.34	< 0.00*
Avoided activities	0.01	0.01	0.29	0.78
Sought reassurance	0.23	0.01	4.94	< 0.00*
Procrastinated	–0.42	0.01	–9.62	< 0.00*
Avoided people	0.29	0.01	6.25	< 0.00*
Energetic	–0.11	0.01	–2.40	0.02*
Enthusiastic	0.08	0.00	1.62	0.11
Accepted or supported	0.10	0.01	2.20	0.03*
Positive	0.01	0.01	0.15	0.88
Content	0.10	0.01	2.17	0.03*

* indicates significance at *p* < 0.05.

## Discussion

The current study uses an observational N-of-1 case study design on intensive repeated digital measures data to investigate the nature of change throughout the course of a modularized therapy. Analysis of the intensive repeated measures data revealed improvements in the pre-therapy period, providing additional evidence for Howard’s remoralization theory (1993). This theory states that the first of three sequential changes throughout the course of psychotherapy is an improvement of the patient’s sense of well-being (*remoralization*), and typically occurs quickly in response to setting up an appointment, getting advice, and other occurrences that tend to happen prior to and at the onset of psychotherapy, including the work done within the first three sessions. Previous work in a variety of treatment settings has found support of this theory, including early gains in optimism very early in a depression treatment study (Schwartz, 1997), and early increases of subjective well-being, followed by reduction of symptom distress, in a study applying the phase model to short-term psychodynamic psychotherapy (Hilsenroth et al., 2001). In the present study, during the first 30-day monitoring period, the participant exhibited significant decreases in negative affect and accompanying significant increases in positive affect. This suggests that the survey paradigm employed in the present study—which might be considered a form of self-monitoring—may serve as a first-step intervention, capable of improving well-being. Extant work has also found support for self-monitoring as a first step in behavior change (Spates and Kanfer, 1977). This understanding that improvement in symptoms can result solely from self-monitoring is important to the first therapy sessions with a patient, and may provide evidence that ecological momentary assessment techniques used prior to therapy may have an intrinsic therapeutic effect in and of themselves.

Pertaining to the module hypotheses, support was found for some, but not all, of our initial hypotheses. After the mindfulness module, it was predicted that restlessness would decrease, yet findings supported change in the opposite direction than hypothesized; after delivery of the module, the participant reported significantly *increased* feelings of restlessness. However, we should note that this increase accounted for only 4% of the variance in restlessness, leaving 96% unexplained. Thus, this increase may be secondary to other, predominant phenomena. Nevertheless, one explanation for this finding is that we did not assess whether or not the participant continued to use the mindfulness exercises following this module, and perhaps they did not incorporate the mindfulness work into any more of their treatment. A second explanation may be that restlessness can occur as one tries to quiet the mind in the beginning stages of meditation; a study investigating the effects of an MBSR on nurse stress and burnout similarly found that, while the program was overall effective at reducing stress, when the participants were asked about the challenges of the program the most common response was restlessness, which was mentioned by 52% of the nurses, with comments such as, “my mind is everywhere,” “my body feels restless,” and “it’s so hard to concentrate!” (Cohen-Katz et al., 2004). Perhaps instituting a more frequent mindfulness practice following this module could mitigate these issues in future work.

Our hypotheses following the exposure module were supported. Significant reductions were observed in difficulty concentrating, avoiding activities, and procrastinating following delivery of the module, illustrating actual changes in the hypothesized downstream targets of the intervention. Of note, endorsement of reassurance seeking significantly *increased* over time following the exposures module, however, we hypothesize that this may be due to the participant conceptualizing reassurance seeking as a pro-social quality rather than a safety behavior. Support was not found for our hypothesis following delivery of the cognitive appraisal and reappraisals module, which we believe may be due to the fact that the predicted targets of worry, depression, positivity, and contentedness were too broad, and that future work should include more specific questions aimed at assessing the success of this intervention (e.g., questions aimed at assessing catastrophizing, overconfidence, and flexible thinking). As noted in the results, an exploratory analysis that examined changes in these constructs over the complete treatment period revealed significant change—in the expected direction—for each, demonstrating that these variables were affected by the treatment generally. Lastly, our hypothesis that worry would decrease after the emotion driven behaviors and emotional avoidance module was supported, illustrating that targeting avoidance in this one individual subsequently improved their worry over the course of this treatment.

In addition to the individual hypothesized changes and subsequent findings, the overall treatment prescription for this individual participant was successful at reducing her symptoms of major depressive disorder and generalized anxiety disorder. The participant began therapy with HAM-A and HAM-D scores of 17 and 11, respectively, indicating that the participant was in the mild severity range of both depressive and anxious symptomatology. One week after the participant completed therapy, her scores for the HAM-A and HAM-D were 4 and 3, respectively, indicating that she fell in the normal ranges for both assessments. Furthermore, separate linear regressions for each survey items as a function of time were conducted over the entire therapy period. The results (Table 6) show significant reductions for the majority of negative affect items, and significant increases for many positive affect items. The participant therefore, upon self-report of the surveys, felt a reduction in negative affect and increase in positive affect throughout therapy.

### Limitations

Limitations of the present study include the use of a single participant, as well as utilizing a method of assessing change per specific module. The changes may have been due to overall changes across the entire therapy period and not due to the specific intervention taking place. Our method of subsetting the data attempted to minimize this from occurring. Other time series designs, including multiple baseline and interrupted time series, may be useful for addressing these questions in the future; since we were performing secondary analyses to a primary study that did not employ these types of designs, we were not able to utilize one here.

### Future Directions

Idiographic approaches to treatment research are growing in popularity. In order to develop more efficient and targeted interventions, researchers and clinicians alike have called for idiographic hypothesis testing to investigate mechanisms of change within individuals over the course of a treatment period. This approach is not new to clinicians, as evidenced by existing conceptualization methods that integrate different modalities to meet the needs of the presenting problems of the individual client, including case formulation driven approaches for cognitive behavior therapy (Persons, 2012), and psychotherapy integration approaches (Stricker and Gold, 1996). Many recent research groups have demonstrated the utility of such approaches, primarily investigating quantitative changes to investigate whether alterations on certain treatment parameters or symptoms predict subsequent changes over time (Brown et al., 2019). This work provides yet another important avenue by which to investigate treatment changes idiographically, serving as a model for a quantitative approach.

It is important to note that while conducted on a single individual, this work was analyzed ideographically, and thus findings are not meant to generalize to other individuals, but rather are meant to illustrate how idiographic work such as this can be utilized perhaps for prediction models (i.e., used to improve prediction of response in the future for that specific N-of-1 unit). Previous inferences made from psychological and medical research (e.g., treatment development, personality research) are typically drawn from statistical tests conducted on aggregated, group-level data, with the implicit assumption that group-level inferences, or findings, will generalize to the individuals who comprise those groups. Often overlooked in this assumption is the problem of ergodicity. Broadly speaking, ergodicity refers to a process by which individual variation can be inferred from group-level data. Historically, the field of psychology has assumed that most processes are ergodic in nature. But this assumption is not always upheld, and recent work by Fisher et al. (2018) found that in self-reported emotion data (and other types of data) the processes were not ergodic. In fact, they found that the variance at the individual level of analysis was up to four times larger than at the group level. Assuming ergodicity for non-ergodic processes leads to misinterpretations of findings that stall the pace of progress in the field. Idiographic work such as this can help to mitigate this gap and provide a groundwork for personalized prediction models.

Furthermore, as noted in the introduction, some researchers believe that therapeutic elements of therapy are due to common factors across all techniques, not specific interventions themselves. Our approach, and supporting evidence, however illustrates the ability to discern specific effects attributed to certain interventions.

The methods employed and findings indicated here also have the potential to aid psychotherapists in routine care by helping them assess whether their interventions are working. By employing routine progress monitoring, whether through daily phone surveys or other methods such as one-time daily diaries, psychotherapists can visualize reductions in symptoms over time and assess whether they are targeting the symptoms they hope, and thus conclude that their prescribed treatment course is effective, or if they need to change course. Methods to collect intensive repeated measures prior to therapy delivery have already been employed in naturalistic settings such as a University health center (e.g., the UC Berkeley Psychology Clinic). These therapists could continue to collect similar data and employ the methods outlined here in order to assess the efficacy and accuracy of their interventions.

## Conclusion

In conclusion, future work should continue to utilize digital health tools to administer quantitative surveys, such as this, as well as other methodologies (e.g., multiple baselines and interrupted time series designs) in order to better understand the nature of change in psychotherapeutic treatment.

## Data Availability Statement

The datasets generated for this study are available on request to the corresponding author.

## Ethics Statement

The studies involving human participants were reviewed and approved by the Committee for Protection of Human Subjects (CPHS) by the Office for Protection of Human Subjects (OPHS) at the University of California, Berkeley. The patients/participants provided their written informed consent to participate in this study.

## Author Contributions

All authors contributed to the work presented in this manuscript. AA and AF designed the experiment and subsequent analyses. LS helped with hypotheses and data organization. AA ran the statistical analyses, analyzed the output data, created the tables and figures, and contributed to the writing of the manuscript.

## Conflict of Interest

The authors declare that the research was conducted in the absence of any commercial or financial relationships that could be construed as a potential conflict of interest.
